# Relationships between body dimensions, body weight, age, gender, breed and echocardiographic dimensions in young endurance horses

**DOI:** 10.1186/s12917-016-0846-x

**Published:** 2016-10-10

**Authors:** D. S. Trachsel, A. Giraudet, D. Maso, G. Hervé, D. D. Hauri, E. Barrey, C. Robert

**Affiliations:** 1 CIRALE-Hippolia, Médecine Sportive, RD 674, F-14430 Goustranville, France; 2 Université Paris-Est, Ecole Nationale Vétérinaire d’Alfort, 7 avenue du Général de Gaulle, F-94704 Maisons-Alfort, France; 3 Office Fédéral de la Statistique, Espace de l’Europe 10, CH-2010 Neuchâtel, Switzerland; 4 INRA, GABI-UMR1313, 78350 Jouy-en-Josas, France

**Keywords:** (3-10): equine, Echocardiography, Cardiovascular, Non-invasive cardiac assessment, Endurance, Body weight, Body dimensions, Gender, Age, Breed

## Abstract

**Background:**

The heart’s physiological adaptation to aerobic training leads to an increase in heart chamber size, and is referred to as the Athlete's heart. However, heart dimensions are also related to body weight (BWT), body size, growth and (in some species) breed. There are few published data on the relationships between heart dimensions and growth or aerobic training in Arabian and Arabian-related endurance horses. Therefore the objective of the present study was to describe the influence of body dimensions (body length (BL), thoracic circumference (TC), withers height (WH)), BWT, age, gender, breed (*purebred Arabians, part-bred Arabians, Anglo-Arabians, and Others*) and the initiation of endurance training on echocardiographic measurements in competition-fit endurance horses aged 4 to 6 years.

**Results:**

Most left atrial (LA) and left ventricular (LV) dimensions increased with age, whereas LA and LV functional indices did not. Although there was no gender difference for LV dimensions, females had larger LA dimensions. In terms of breed, *Anglo-Arabians* had the largest LV dimensions. Regression models indicated that the included explanatory factors had a weak influence on heart dimensions. Age, body dimensions, breed and gender showed the most consistent influence on LA dimensions, whereas BWT, breed and kilometres covered in competition showed the most consistent influence on LV dimensions.

**Conclusion:**

The increase in echocardiographic dimensions with age indicates on-going growth in our population of 4 to 6 year-old horses. We also observed small changes associated with the initiation of endurance training. Morphometric dimensions had a greater influence on LA dimensions, whereas LV dimensions were also influenced (albeit weakly) by parameters associated with exercise intensity. These results may therefore reflect early adaptations linked to the initiation of endurance training.

**Electronic supplementary material:**

The online version of this article (doi:10.1186/s12917-016-0846-x) contains supplementary material, which is available to authorized users.

## Background

The Athlete's heart is a physiological adaptation to aerobic training and athletes (especially endurance athletes) have larger hearts and thus a larger stroke volume than non-endurance trained or sedentary humans [1, 2]. The Athlete's heart has also been described in various types of racehorse and endurance horses [3–7] and has been linked to the appearance of heart murmurs albeit with little impact on performance [6, 8–10]. Furthermore, a high VO_2max_ (an index of exercise capacity) in horses has been related to larger left ventricular (LV) dimensions and LV mass [11, 12]. Larger hearts have been associated with better performance in some studies, [4, 5, 13] but not in others [14] and the discrepancy between these studies can certainly be explained by varying parameters used to assess performance. It is nevertheless the case that in most equestrian sport disciplines, horses that train more, race more, earn more or participate in elite competitions have larger hearts than their peers [4, 5, 7, 13].

However, other factors (such as body dimensions, body weight (BWT) [5, 6, 15], breed [16, 17] and gender [5, 6, 13]) might influence the heart’s dimensions. Some of the studies in this field included non-fully mature horses [3, 5, 13], whereas others also included adult horses [6, 7]. Especially in young individuals, growth is an important matter, and age at maturity differs from one breed to another [18–21]. Furthermore, training methods and the physical demands of competition differ from one discipline to another. Thus, conclusions based on data from one breed and/or discipline cannot necessarily be fully extrapolated to another breed and/or discipline. Hence, the primary objective of the present study was to describe the development of the heart in young Arabian-type horses aged between four and six years, an age at which endurance training is usually initiated. The second objective was to assess a possible association between external factors and the echocardiographic dimensions of the heart’s chambers and great vessels in these horses.

## Methods

### Population

Between 2011 and 2014, echocardiographic measurements were recorded during field exercise tests and at the annual finals of the French National Championships for young endurance horses. The field tests were organised by the research group for all interested endurance horses owners four times a year. Only horses aged between 4 and 6 years at the time of presentation and with at least one purebred Arabian parent were included in the study. All horses were examined by the official veterinarians on site and had thus been authorised to race prior to enrolment in the study. Each horse was identified by its French national identification number and was included only once in our subsequent analyses. The study design was approved by the local institutional Ethical Committee (ComEthANSES/ENVA/UPEC, Maisons-Alfort, France; approval number: 12/07/11-1). All owners provided their written, informed consent to participation before the horses underwent any study procedures and were told that they could withdraw their horse from the study at any time.

For each horse, body length (BL, in cm: the distance between the shoulder at the palpable portion of the greater tubercle and the palpable portion of the ischial tuberosity), thoracic circumference (TC, in cm: circumference measured at girth placement), withers height (WH, in cm: height at the highest point of the withers) were measured with a tape measure. The BWT (in kg) was measured using a portable horse-weighing platform (Horse weigh® Tokyo model, Horse Weigh Ltd, Llandrindod Wells, UK, http://www.horseweigh.com). The body surface area (BSA, in m^2^) [22] was calculated using the following equation: BSW = 0.101 × BWT^2/3^. Horses were divided into age groups: *Group 4y* included all 4-year old horses, *Group 5y* included all 5-year old horses, and *Group 6y* included all 6-year old horses. Each horse’s gender and breed were also recorded. Although all the horses necessarily had a least one purebred Arabian parent (given the study’s inclusion criterion), they were divided into four breed groups for further analysis: *Group purebred Arabians* comprised all the purebred Arabians and the Shagya Arabians, *Group part-bred Arabians* comprised crossbreeds between Arabians and Warmbloods, *Group Anglo-Arabians* comprised Anglo-Arabians or Anglo-Arabian crossbreed, and lastly, *Group Others* comprised all other Arabian crossbreeds.

Given that the owners of the included horses applied a very broad variety of training programmes, it was not possible to objectively quantify the horses’ training history. However, a rough estimation of each horse’s activity prior to echocardiography was derived by noting the total number of kilometres covered in competition since the first registered competition (Km-career) and the numbers of days since the first registered competition (d-career) from the records in the French Equestrian Federation’s database (FFECompet https://ffecompet.ffe.com/cheval [23]).

### Echocardiographic measurements

Transthoracic, two-dimensional echocardiography (2DE) was performed on resting horses before they participated in the endurance competitions or field exercise tests. The echocardiography was performed by two experienced clinicians (AG for the 68 examinations in 2011, and DST for the 272 examinations in 2012-2014) using a portable ultrasound system (Vivid I, General Electric Healthcare Europe GMBH, France) that enabled synchronous recording of the electrocardiogram (ECG). The field conditions and the limited time available for the examinations precluded the realisation of Doppler ultrasound examinations and allowed only recordings from the right parasternal long axis views, as described elsewhere in detail [24–27].

The measurements were made under supervision of the experienced clinicians by two well-trained persons independent of those who examined the horses (DM, GH). To summarise, the dimension of the aortic (sinus) diameter (AOD), and the maximal pulmonary artery diameter (PAD) were measured at end-diastole on the right parasternal long-axis 2 DE views of the left and right ventricular outflow tract, respectively. The atrial dimensions were assessed in a right parasternal long-axis 2 DE view centred on the LA by measuring the maximal LA diameter (LAD_max_), and LA area (LAA_max_) at end-systole prior to mitral valve opening, the LA diameter (LAD_a_) and LA area (LAA_a_) at the onset of the electrocardiographic P wave prior to active contraction, and the minimal LA diameter (LAD_min_) and LA area (LAA_min_) at the time of mitral valve closure. The LV dimensions were also assessed in a right parasternal long-axis 2DE view but centred to visualize the LV apex. From this view, the interventricular septal thickness (IVS), the LV internal diameter (LVID), and the LV free wall thickness (LVFW) were measured on a line midway between the papillary muscles and the mitral valve annulus, perpendicular to the IVS and LVFW and parallel to the mitral valve annulus at end-diastole (d) and peak systole (s). Using the same view LV internal area (LVIA) and LV internal length (LVIL) were measured at end-diastole (d) and at peak systole (s).

The LA areas were used to calculate functional indices of LA contraction including the active LA fractional area change [Active LA FAC = (LAA_a_ – LAA_min_)/LAA_a_ × 100], the passive LA fractional area change [Passive LA FAC = (LAA_max_ – LAA_a_)/LAA_max_ × 100], the total LA fractional area change [Total LA FAC = (LAA_max_ – LAA_min_)/LAA_max_ × 100], and the active-to-total LA area change [active:total LA AC = (LAA_a_ – LAA_min_)/(LAA_max_ – LAA_min_)] [25].

For measured LV dimensions, the relative wall thickness at end-diastole [RWT = (IVS_d_ + LVFW_d_)/LVID_d_], the mean wall thickness at end-diastole [MWT = (IVS_d_ + LVFW_d_)/2] and the LV_mass_ [1.04×[(LVID_d_ + LVFW_d_ + IVS_d_)^3^-LVID_d_]-13.6] were calculated.[5, 11, 24]. Two functional indices for the LV were calculated: the LV fractional shortening [LV FS = (LVID_d_ – LVID_s_)/LVID_d_ × 100]) and the LV fractional area change [LV FAC = (LVIA_d_ – LVIA_s_)/LVIA_d_ × 100] [5, 11, 24, 27].

Further, the cardiac output [CO = SV × HR] and the stroke volume [SV = LVIV_d_ – LVIV_s_], based on LV internal volume at end-diastole (LVIV_d_) and at peak systole (LVIV_s_), were calculated using the equation 5/6×LVIA^2^/LVIL (describes as the "4CAL equation" by McConachie et al. [28]).

In order to compare the relative proportions of the heart’s chambers, the LAA_max_/LVIA_d_, and LAD_max_/LVID_d_ ratios were calculated. Further, the AOD/PAD ratio was established.

All measurements were made offline on the basis of digitally stored cine-loop recordings and were averaged over two or three non-consecutive cardiac cycles. The heart rate (HR) was determined and the rhythm was assessed on the ECGs recorded synchronously on the cine-loops.

### Statistical analyses

Based on a visual assessment of histograms and of normal probability plots, normality of the data distribution was given and parametric methods were applied.

Influences of age category, gender and breed were tested using an analysis of variance (ANOVA) with Holm-Sidak post-hoc tests for multiple comparisons. Further, pooled males (geldings and intact males) were compared with females using a Student’s *t*-test. Dichotomous variables were compared in a Chi^2^ test. The threshold for statistical significance was set to *p* < 0.05.

Univariate regression models were established for LA and LV echocardiographic measurements and for explanatory factors with continuous data (BWT, BSA, WH, TC, BL, Km-career, d-career). Multivariate regression models were then developed for the most relevant LA and LV measurements. Based on an assessment of the residuals, log-linear regression models were used if necessary. The log transformation of the outcomes allowed reaching normality of the residuals. All explanatory factors were included in the initial model. Thereafter, quadratic and cubic terms were also included for the BWT in the initial models. Non-relevant explanatory factors were excluded by stepwise elimination based on the adjusted R^2^ and the Akaike information criterion (AIC). However, models were not considered to be relevant if the final adjusted R^2^ was below 10 %. No interactions between explanatory factors were considered in the models.

All statistical analyses were performed using commercially available software (GraphPad Prism®, version 6.05, GraphPad Software, San Diego, CA, USA, www.graphpad.com, SigmaStat®, version 3.5, Systat Software GmbH, Erkrath, Germany, R-software®, version 3.1.0, R Development Core Team, Vienna, Austria).

## Results

Three hundred and forty horses were included in the study. The demographic characteristics of the study population are summarised in Table 1. Given that the field conditions limited the available time per horse, the number of missing data varied from one measurement to another. The numbers of available data per measurement are given in Additional files 1, 2, 3 and 4. Most horses were females or geldings, and intact males were significantly underrepresented (Chi^2^ *p* < 0.01). Furthermore, most of the horses were *purebred* or *part-bred Arabians*, whereas *Anglo-Arabians* and *Others* were significantly underrepresented (Chi^2^ *p* = 0.03). There were slightly more horses in *Group 6y* than in the two other groups. The measured HR was slightly higher than the usual resting HR for horses, and no arrhythmias were observed on the reviewed cine-loops.

**Table 1:** Demographic characteristics of the study population

	4-year-olds	5-year-olds	6-year-olds	Total	*p* ^#^
Number	101	109	130	340	n.s.
	Mean (± SD)	Mean (± SD)	Mean (± SD)	Mean (± SD)	
HR (bpm)	44 (±10)	44 (±9)	43 (±10)	43 (±9)	n.s.
BWT (kg)	405.9^a^ (±39.2)	422.9^b^ (±35.3)	411.1^a^ (±37.0)	413.4 (±37.6)	*
BSA (m^2^)	3.9^a^ (±0.2)	4.0^b^ (±0.2)	3.9^a^ (±0.2)	3.9 (±0.2)	*
WH (cm)	152.9 (±3.8)	153.5 (±4.0)	152.5 (±4.3)	152.9 (±4.0)	n.s.
TC (cm)	173.5 (±7.6)	174.7 (±6.8)	173.1 (±5.8)	173.8 (±6.7)	n.s.
BL (cm)	154.2 (±7.3)	154.7 (±7.5)	152.9 (±7.4)	153.9 (±7.4)	n.s.
d-career (days)	109.7^a^ (±69.5)	302.5^b^ (±175.1)	576.3^c^ (±268.8)	351.5 (±276.5)	**
Km-career (km)	44.3^a^ (±49.7)	130.5^b^ (±82.8)	435.8^c^ (±323.6)	223.0 (±269.8)	**
Gender					
females	42	52	64	158	**
geldings	34	36	48	118	
intact males	25	21	18	64	
Breed					
Purebred Arabian	49	64	88	201	*
Part-bred Arabians	39	30	31	100	
Anglo-Arabians	5	11	8	24	
Others	8	4	3	15	

^#^, results from ANOVAs or Chi^2^ tests, as appropriate; *, significant at *p* < 0.05; **, significant at *p* < 0.01; n.s., not significant; results marked with a different superscript letter differed significant from the other results in the row. For *p*-values, see Additional file 5

The p-values obtained in ANOVAs for age groups, genders and breed groups are presented in Additional files 5, 6 and 7. In summary, the BWT and the BSA were significantly greater in 5-year old horses than in 4-year or 6-year old horses. The body dimensions in *Group 5y* were also slightly (but not significantly) higher than those in the other two age groups.

Further, the horses in *Group 6y* had significantly longer careers and had covered significantly more kilometres in competition than those in the other age groups. Likewise, values for Km-career and d-career were also significantly higher in *Group 5y* than in *Group 4y.*

The two-dimensional LA dimensions increased fairly steadily with age and significant differences were noted when comparing *Group 4y* with *Group 5y* or *Group 4y* with *Group 6y* (Additional file 6). However, the largest difference was observed when comparing *Group 4y* and *Group 6y*. LA area measurements also increased with age, although the differences between the age groups were not significant. The LA functional indexes did not differ significantly with age. *Group 5y* and *Group 6y* did not differ significantly in terms of any of the LA dimensions.

AOD and AOD/PAD increased constantly from *Group 4y* to *Group 6y*, whereas PAD was lower in *Group 4y* than in *Group 5y* (Additional file 6).

Most LV echocardiographic measurements (Additional file 7) also increased with age and the largest differences were observed when comparing *Group 4y* and *5y.* The differences between *Group 5y* and *6y* were less pronounced but were most marked for two-dimensional measurements. In contrast, increases in area measurements were more regular over the studied age range. *Group 5y* and *6y* did not differ significantly in terms of any of the LV measurements. We did not observe any age-related changes for IVS_s_, IVS_d_, LVFW_s_, LVFW_d_, MWR, RWT, LV FS, LV FAC and CO.

Overall, gender differences were small (Additional files 5, 6 and 7). The comparison between genders showed that females had a larger TC than intact males or pooled males. Geldings had slightly greater WH and BWT than the other groups, and intact males had the lowest values for all morphometric dimensions; however, the differences were not statistically significant. There were no gender differences in Km-career or d-career. Intact males had the smallest two-dimensional LA dimensions, whereas females had the largest LA area measurements (Additional file 6). These differences in dimensions led to a significant gender difference for one of the LA functional indices: the passive LA FAC was highest in intact males. There were no significant differences for other comparisons. With regard to the great vessels, the AOD was slightly (but not significantly) lower in females. The PAD was significantly greater in females when compared with geldings or with all males. Hence, the ratio AOD/PAD was also smaller in females than in geldings or in all males. There were no gender differences in the LV measurements (Additional file 7).

There were no differences for Km-career or d-career between the breed groups. In our population *purebred Arabians* were smallest and lightest, whereas *Anglo-Arabians* were tallest and heaviest (Additional file 5). Further, none of the differences in LA dimensions between breed groups were statistically significant (Additional file 6). However, two-dimensional LA measurements were highest in *Anglo-Arabians*, whereas in *part-bred Arabians* and *Other*s these dimensions were slightly smaller than in *purebred Arabians*. The same difference was not apparent for area measurements, and *purebred Arabians* even showed a trend towards highest values. There were no obvious differences in AOD or in PAD between the breeds.

As seen for the LA, all LV measurements were highest in *Anglo-Arabians* and for most measurements lowest in *part-bred Arabians* and *Others* (Additional file 7). The largest differences were observed for measurements made at end-diastole, and were statistically significant for IVS_d_, LVID_d_, LV_mass_ and SV.

Univariate linear regression models revealed very weak influences of the explanatory variables on echocardiographic measurements (Additional files 1 and 2). Less than 14 % of the total variability in the LV dimensions (LVIA_d_: R^2^ = 11.0 %; LVIV_d_: R^2^ = 12.4 %; SV: R^2^ = 12.6 %) and in the LV_mass_ (R^2^ = 13.7 %) was explained by the BWT or BSA. Less than 11 % of the total variability of the LA measurements was explained by TC (LAD_a_: R^2^ = 11.0 %; LAA_max_: R^2^ = 11.0 %).

In the multivariate regression models, the values for the adjusted R^2^ were also low and the chosen explanatory factors accounted for less than 18 % of the total variability in the echocardiographic measurements (Additional files 3 and 4). Furthermore, the analyses for LA and LV measurements showed different patterns. For LA measurements, the values for the adjusted R^2^ were higher than those obtained for LV measurements, and most of the LA measurements were related to morphometric measurements.

In these multivariate regression models, BL showed a positive influence on LAD_max_, BL and TP showed a positive influence on LAD_a_ and AOD. BL and WH had a positive influence on LAD_min_. LAA_a_ and LAA_min_ increased with increasing WH, TP and BL, whereas LAA_max_ increased only with increasing TP and WH. BWT had positive influence only on LAD_max_, AOD and PAD. BSA is not reported as results were identical to those for BWT.

For most LA measurements, age positively influenced the dimensions. Hence older horses displayed larger dimensions, except for the PAD, which decreased with age.

Although *Anglo-Arabians* showed a trend towards larger two-dimensional LA measurements, breed had a negative influence on LAD_max_, LAA_a_ and LAA_min_ in the multivariate regression models and belonging to a breed other than *purebred Arabians* was associated with a decrease in the mentioned LA dimensions.

As seen in the ANOVAs, multivariate regressions showed that gender was variously associated with the magnitude of some LA measurements. Intact males and geldings showed lower LAD_a_, LAD_min_, LAA_a_ and LAA_min_ values than females did. Intact males and geldings had smaller PADs but higher AODs than females. The Km-career and d-career variables were not associated with the LA measurements. The PAD increased with Km-career and decreased very slightly with d-career.

For LV measurements (Additional file 4), most measurements were positively associated with BWT. This was especially true for LV_mass_, LVIA_d_, and derived values such as LVIV_d_ and SV. When considering other morphometric measurements, WH had positive influence on LVIA_d_, LVIV_d_ and SV, and BL had a negative influence on LVIA_d_, LVIV_d_ and SV. The results for BSA are not reported in detail because they were identical to those for BWT.

In comparison to *purebred Arabians,* LV_mass_ was higher in the *Anglo-Arabians* and lower in the *part-bred Arabians*. Gender and age had only a slight influence on LV measurements. However, Km-career was positively associated with LV_mass_, LVIA_d_ and LVIV_d_.

## Discussion

The present study reports changes in echocardiographic dimensions of the heart in young Arabian type horses at the end of their growth period and at the beginning of their endurance training for competitions. All external variables included in the analyses influenced to some extent several of the measured dimensions. Morphometric dimensions, age and breed showed most consistently an influence on LA measurements, whereas BWT, breed and Km-career had most consistently an influence on LV measurements.

All the horses in the study population had at least one purebred Arabian parent. Our study population was chosen to be representative of the horses participating in endurance competitions. This choice was in accordance with literature data indicating that purebred or mixed-breed Arabians are largely overrepresented in endurance competitions, although some other breeds do participate [29, 30]. In view of the inclusion criteria, the division into several breed groups may seem arbitrary. However, there were significant differences between these breed groups. *Purebred Arabians* were the smallest and lightest - giving them advantage over other breeds in endurance competitions [29, 30]. Body dimension and BWT were highest in *Anglo-Arabians* that in turn led also to larger LV_mass_. Nevertheless, in the multivariate regression models, belonging to breeds other than *purebred Arabians* was associated with smaller LA measurements. This indicated that the smallest horses in our population did not necessarily have the smallest hearts and hence that other factors must be considered.

BWT and body dimensions were higher in *Group 5y* than in *Group 4y*, which was presumably due to on-going growth. In contrast, the smaller body dimensions in *Group 6y* (especially WH and BL, which are less influenced by body condition) might be due to the selection of smaller, lighter, more compact horses for more demanding competitions. Indeed, high BWT in endurance horses has been associated with failure to finish competitions, particularly due to lameness [29, 30]. Furthermore, horses in *Group 6y* had covered a greater distance in competition (Km-career) and thus were certainly subjected to harder training, which may have resulted in a lower BWT. Training reduces fat content and increases muscle mass. Therefore, it would have been interesting to measure lean body weight. BWT and especially fat-free mass were associated with better indices of performances in Standarbreds, [31, 32] and fat-free mass was also associated with a higher VO_2max_. However, too low BWT or too low percentage of body fat is detrimental in human athletes [33, 34]. The optimal percentage of body fat has not yet been established for horses, although body fat was around 8 % in well-performing athletic horses [29, 32, 35]. As is the case for human athletes, the optimal percentage of body fat doubtless varies from one discipline to another [34, 36].

All heart dimensions increased with age. The difference between 4 and 5 years of age was greater than the difference between 5 and 6 years of age. In univariate and multivariate regression analyses, for most heart dimensions a weak relationship was observed with BWT or TC (especially for LA measurements). The higher values for the R^2^ in regression models of LA measurements and of the dimensions of the great vessels, and their relationship with body dimensions (rather than BWT) might indicate that these dimensions are more strongly related to the horse’s morphotype. Al-Haidir et al. also have reported that some echocardiographic measurements are somewhat more strongly associated with TC than they are with BWT [37]. However, due to the present study’s inclusion criteria, the range of body dimensions and weights was narrower than in the work by Al-Haidir et al. [37]. Concomitant increases in all dimensions are seen with growth, and growth has to be taken into consideration when studying young individuals. Different breeds have differing growth rates. Thoroughbreds and Standardbreds are fast-growing breeds and reached 83 % of the adult BWT by the age of 2 years, whereas the growth rate is slower in other breeds and by the age of 2 years Warmbloods, Anglo-Arabians and Lusitanos reached 80 %, 75 % and 77 % of their adult BWT, respectively [20, 21]. Arabians have been classified as slow-growing horses [38]. Accordingly, they are still growing between the ages of 4 and 6 years. This contrasts with Standardbred horses, which are considered to reach maturity between the ages of 3.5 and 5 years [6]. Although growth certainly affects the LV dimensions, the latter are also greatly modulated by other factors. In particular, training is a strong stimulus for cardiac hypertrophy, and we detected a weak but significant association between Km-career and some of the LV measurements. This very weak association might be due to the poorly elaborated performance parameters (Km-career, d-career) and also due to the generally low level of endurance training seen at this age [39]. A training-related increase in LV dimensions has been observed in Thoroughbreds [3], Standardbreds [5, 6] and elite Arabians [7]. The absence of a change in RWT indicated that the heart’s adaptation to exercise in our population is more similar to that seen in Standardbreds [5, 6] than to that seen in Thoroughbreds, [3] or elite Arabians [7]. Further, this finding might also illustrate differences in training practices among different breeds. We did not find any association between LA dimensions and Km-carrer. This is in contrast to studies in human athletes that showed also greater LA dimensions in presence of an Athlete’s heart [40, 41]. However, the endurance training in our study population was probably not intensive enough to induce changes in LA dimensions.

The results of our regression analyses agree with another study reporting small values for the R^2^ for LV measurements in young (2-year-old) Standardbreds [6]. However, values for the R^2^ increased with age in this previous study, particularly for LV_mass_. This comparison also suggests that our population’s training was not yet hard enough to induce marked changes in heart dimensions.

In accordance with previous studies [42–44], there were no age-related differences in LA and LV functional indexes.

Females had the largest TC. Given that LA measurements were related to TC in our regression models, we were not surprised to find that females had also the largest LA measurements. On the contrary, intact males had the smallest LA dimensions. In contrast to other studies [5, 13, 44], gender did not appear to influence LV dimensions in our study population. One explanation could be that the intensity of the aerobic training in endurance horses at this age is not high enough to bring out the full potential for hypertrophy in intact males. Another explanation might be related to the custom of French endurance trainers/owners to breed females at the age of 5 years. Gestation may have induced changes in heart dimensions (as seen in humans and dogs), although these changes are usually reversible [45, 46].

The present study had a number of limitations. Due to field conditions and the limited time available per horse, not all echocardiographic views of heart chambers could be taken, and some views were of poor quality. Similarly, it was not possible to perform Doppler examinations and thus screen for possible regurgitations. Due to these technical issues there were several missing data. Nevertheless our analyses were based on a large body of data. Given that the measured heart chamber dimensions can be influenced by the transducer position and the views obtained [47, 48], not all of our measurements can be validly compared with the literature values. Our comparison with previous studies is therefore limited to the consideration of factors that influence heart dimensions. Second, the use of only longitudinal views meant that our LV measures could probably be underestimated [47]. Furthermore, the measured HRs were slightly over resting values, and the shorter filling time at these HRs might have affected ours results, especially for diastolic measurements. Nevertheless, the HRs measured in our studies were only slightly higher than those reported in the literature for endurance horses [7, 48]. Lastly, the fact that the recordings and measurements were made by several operators probably decreased the precision of our measurements [25, 49].

Differentiating the effects of growth from the effects of training would have required the use of a control group. In principle, a control group should comprise untrained, non-competing horses of the same age. Unfortunately, this type of population was not available in our research setting. Furthermore, follow-up measurements of our study population 3-4 years later or measurements from a control group of mature, elite endurance horses would have provided information on the effects of more intensive aerobic training. In accordance with results from Sleeper et al. [7] reporting greater LVID, LV_mass_ and SV in elite Arabians (mean age 21.1, age range 7-17) than in non-elite Arabians, data from our research group show that in a smaller group of endurance horses (*n* = 11; nine purebred Arabians and two others; four females, six geldings and one intact male; mean age ten, range 8-13 years) participating in races of least 120 km, the main differences relative to younger horses were seen for LVID, LVIA, LVIL and LVIV, with greater differences appearing in peak systolic measurements. Furthermore, in the older horses, LV_mass_ was higher and LV FAC and LV FS were lower. The greater LV dimensions and LV_mass_ agree with literature data on various breeds [4, 6, 7] and are certainly related to a greater aerobic training volume at this age. These considerations strengthen our hypothesis whereby the training load in younger horses was not intense enough to induce marked cardiac hypertrophy. Our findings with regard to LV FAC and LV FS are more difficult to explain, since the Athlete’s heart does not feature any changes in LV function at rest [1, 41]. The results might be best explained by the fact that older horses were more used to being handled and therefore showed a narrower range of heart rates than younger horses. Indeed, the HRs for adult endurance horses reported in the literature were slightly lower than in the present study [7, 48].

Furthermore, the performance parameters used in the present study (Km-career, d-career) were very rough. In France, purebred Arabians are not trained intensively between the ages of 4 and 6 years and training is not as standardised as it is in Thoroughbreds or Standardbreds [39]. Furthermore, some horses were bred during this period, which decreased the training period. Therefore, it was not possible to analyse objective parameters, such as gains, speed at kilometre, or numbers of start [4, 5, 13, 14]. The most accessible parameters were Km-career and d-career, since this information was freely available in the FEE database. We hypothesised that a horse having participated in a greater number of competitions or longer competitions would have been trained harder. Even though we observed a weak relationship between Km-career and some LV measurements, better performance indicators are clearly needed. Long-term information on the endurance horses’ competitive performance was not yet available at the time of the present study and will be addressed in the future.

Lastly, we had no possibility to record and therefore to compare echocardiographic measurements taken before and after exercise. This comparison would have provided valuable information on cardiac adaptation to effort in competition [50, 51] but was outside the scope of the present study. Hence this topic merits further research.

## Conclusion

In a population of endurance horses aged four to six years, the main changes in cardiac dimensions were observed between age groups and are probably due to on-going growth, even though the initiation of endurance training may also have had some effect. In particular, LV measurements were related (albeit weakly) to performance parameters, which might reflect the adaption to the initiation of aerobic training. Gender differences were not apparent in our young population, although there were significant differences between breeds. Despite being the smallest horses, *purebred Arabians* did not have the smallest hearts. Small body size might be an advantage in endurance competitions. However, other factors, such as the heart’s dimensions relative to the body dimension, or the capacity of a rapid HR recovery after the race, appear to be important and should be studied in the future. Further longitudinal studies are needed, in particular to validate specific performance indicators for endurance horses.

## Additional files

Additional file 1: Results of the univariate linear regression analyses, showing the weak influence (expressed as R^2^) of the chosen explanatory parameters on left atrial (LA) and great vessel echocardiographic dimensions. (DOCX 16 kb)
Additional file 2: Results of the univariate linear regression analyses, showing the weak influence (expressed as R^2^) of the chosen explanatory parameters on left ventricular (LV) echocardiographic dimensions. (DOCX 16 kb)
Additional file 3: Results of the multivariate regression analyses, showing the influence of external factors considered as independent variables on left atrial (LA) and great vessel echocardiographic dimensions. (DOCX 29 kb)
Additional file 4: Results of the multivariate regression analyses, showing the influence of external factors considered as independent variables on left ventricular (LV) echocardiographic dimensions. (DOCX 37 kb)
Additional file 5: Results of the ANOVAs and Student’s t-tests reporting p-values for comparisons of morphometric measurements between age groups, genders and breeds groups. (DOCX 17 kb)
Additional file 6: Results of the ANOVAs and Student’s t-tests reporting p-values for comparisons of left atrial (LA) and great vessels dimensions between age groups, genders and breeds groups. (DOCX 18 kb)
Additional file 7: Results of the ANOVAs and Student’s t-tests reporting p-values for comparisons of left ventricular (LV) echocardiographic dimensions between age groups, genders and breeds groups. (DOCX 21 kb)

## Abbreviations

2DE: Two-dimensional echocardiography
active LA FAC: Active left atrial fractional area change
active:total LA AC: Active-to-total left atrial area change
AIC: Akaike information criterion
ANOVA: Analyse of variance
AOD: The dimension of the aortic (sinus) diameter
BL: Body length
BSA: Body surface area
BWT: Bodyweight
CO: Cardiac output
d: At end-diastole
d-career: Numbers of days since the first recorded official competition
ECG: Electrocardiogram
HR: Heart rate
IVS: Interventricular septal thickness
Km-career: Number of km covered in completion since the first recorded official competition
LA: Left atrium
LAA_a_: Left atrial area prior to active contraction, at the onset of the electrocardiographic P-wave
LAA_max_: Maximum left atrial area at end-systole, prior to mitral valve opening
LAA_min_: Minimum left atrial area at end-diastole, after mitral valve closure
LAD_a_: Left atrial diameter prior to active contraction, at the onset of the electrocardiographic P-wave
LAD_max_: Maximum left atrial diameter at end-systole, prior to mitral valve opening
LAD_min_: Minimum left atrial diameter at end-diastole, after mitral valve closure
LV FAC: Left ventricular fractional area change
LV FS: Left ventricular fractional shortening
LV: Left ventricular
LVFW: Left ventricular free wall thickness
LVIA: Left ventricular internal area
LVID: Left ventricular internal diameter
LVIL: Left ventricular internal length
LVIV: Left ventricular internal volume
LV_mass_: Left ventricular mass
MWT: Mean wall thickness
PAD: Maximal pulmonary artery diameter
passive LA FAC: Passive left atrial fractional area change
RWT: Relative wall thickness
s: At peak systole
SV: Stroke volume
TC: Thoracic circumference
total LA FAC: Total left atrial fractional area change
WH: Withers height
