# Supplementary material for: Relationships between body dimensions, body weight, age, gender, breed and echocardiographic dimensions in young endurance horses
Source: BMC Vet Res. 2016 Oct 10;12:226. doi: 10.1186/s12917-016-0846-x (PMC5057441; doi:10.1186/s12917-016-0846-x)
Supplement: Additional file 1: — Results of the univariate linear regression analyses, showing the weak influence (expressed as R2) of the chosen explanatory parameters on left atrial (LA) and great vessel echocardiographic dimensions. (DOCX 16 kb) [file 12917_2016_846_MOESM1_ESM.docx]

Additional File 1: Results of the univariate linear regression analyses, showing the weak influence (expressed as R^2^) of the chosen explanatory parameters on left atrial (LA) and great vessels echocardiographic dimensions.

|  | n | BWT | BSA | WH | BL | TC | Km-career | d-career |
| --- | --- | --- | --- | --- | --- | --- | --- | --- |
| **LA Dimensions** |  |  |  |  |  |  |  |  |
| LAD_max_ | 255 | 6.2 | 6.3 | 4.9 | 6.4 | 5.6 | 1.6 | 0.9 |
| LAD_a_ | 255 | 6.1 | 6.1 | 4.4 | 5.9 | **11.0** | 1.4 | 2.2 |
| LAD_min_ | 255 | 4.4 | 4.5 | 3.6 | 5.0 | 8.8 | 2.6 | 2.9 |
| LAA_max_ | 255 | 5.4 | 5.4 | 7.2 | 4.3 | **11.0** | 2.3 | 1.3 |
| LAA_a_ | 255 | 4.8 | 4.9 | 5.9 | 5.9 | 9.4 | 1.1 | 1.2 |
| LAA_min_ | 255 | 3.9 | 4.0 | 4.9 | 3.7 | 5.1 | 0.2 | 0.9 |
| **LA Functional indices** |  |  |  |  |  |  |  |  |
| active LA FAC | 253 | 0.1 | 0.1 | <0.1 | <0.1 | <0.1 | 0.1 | <0.1 |
| passive LA FAC | 253 | 0.3 | 0.3 | 0.3 | 2.1 | 0.4 | <0.1 | 0.3 |
| Total LA FAC | 253 | 0.3 | 0.3 | 0.2 | 0.7 | <0.1 | 0.1 | 0.3 |
| active: total La AC | 253 | 0.1. | 0.1 | <0.1 | <0.1 | <0.1 | <0.1 | <0.1 |
| **Great vessels** |  |  |  |  |  |  |  |  |
| AOD | 245 | 9.4 | 9.4 | 6.5 | 6.3 | 9.5 | 2.9 | 0.1 |
| PAD | 239 | 1.3 | 1.3 | 2.2 | 0.3 | 6.6 | 3.3 | 2.0 |
| AOD/PAD | 233 | 0.2 | 0.2 | <0.1 | 0.7 | 0.1 | 0.4 | 4.9 |

R^2^ above 10% are highlighted in bold. n, number of measurements available for the analyses; see abbreviation list for meaning of abbreviations for LA and great vessels measurements.
