# Supplementary material for: Relationships between body dimensions, body weight, age, gender, breed and echocardiographic dimensions in young endurance horses
Source: BMC Vet Res. 2016 Oct 10;12:226. doi: 10.1186/s12917-016-0846-x (PMC5057441; doi:10.1186/s12917-016-0846-x)
Supplement: Additional file 2: — Results of the univariate linear regression analyses, showing the weak influence (expressed as R2) of the chosen explanatory parameters on left ventricular (LV) echocardiographic dimensions. (DOCX 16 kb) [file 12917_2016_846_MOESM2_ESM.docx]

Additional File 2: Results of the univariate linear regression analyses showing the weak influence (expressed as R^2^) of the chosen explanatory parameters on left ventricular (LV) echocardiographic dimensions.

|  | n | BWT | BSA | WH | BL | TC | Km-career | d-career |
| --- | --- | --- | --- | --- | --- | --- | --- | --- |
| **LV Dimensions** |  |  |  |  |  |  |  |  |
| IVS_d_ | 266 | 1.9 | 1.9 | 0.4 | <0.1 | 0.2 | 0.3 | 0.8 |
| LVID_d_ | 261 | 3.6 | 3.6 | 2.2 | 0.3 | 0.3 | <0.1 | <0.1 |
| LVFW_d_ | 261 | 2.5 | 2.4 | 0.9 | 1.8 | 2.9 | 4.7 | 0.7 |
| MWT | 257 | 4.5 | 4.5 | 1.4 | 1.1 | 1.5 | 4.6 | 1.9 |
| RWT | 257 | 0.5 | 0.4 | <0.1 | <0.1 | 0.4 | 2.2 | 0.8. |
| LV_mass_ | 260 | **13.7** | **13.7** | 6.6 | 2.6 | 3.4 | 5.6 | 1.7 |
| LVIA_d_ | 317 | **10.6** | **10.6** | 7.7 | 0.4 | 2.7 | 2.9 | 1.3 |
| LVIL_d_ | 317 | 3.5 | 3.5 | 4.4 | <0.1 | 1.1 | <0.1 | 0.6 |
| LVIV_d_ | 312 | **12.4** | **12.4** | 8.2 | 0.8 | 3.3 | 4.1 | 1.4 |
| **LV functional indices** |  |  |  |  |  |  |  |  |
| LV FS | 257 | <0.1 | <0.1 | <0.1 | <0.1 | <0.1 | 0.1 | 0.1 |
| LV FAC | 305 | <0.1 | 0.4 | <0.1 | 0.6 | 1.4 | 1.1 | <0.1 |
| SV | 317 | **12.6** | **12.6** | 6.8 | 1.1 | 4.3 | 1.1 | <0.1 |
| CO | 317 | 3.8 | 3.8 | 3.7 | 0.2 | 2.4 | 0.2 | <0.1 |
| LAA_max_/LVIA_d_ | 240 | 0.7 | 0.7 | <0.1 | 0.3 | 0.7 | 0.1 | <0.1 |
| LAD_max_/LIVID_d_ | 193 | <0.1 | <0.1 | <0.1 | 0.2 | 0.3 | 0.8 | 0.6 |

R^2^ above 10% are highlighted in bold. n, number of measurements available for the analyses; see abbreviation list for meaning of abbreviations for LV measurements.
