# Supplementary material for: Relationships between body dimensions, body weight, age, gender, breed and echocardiographic dimensions in young endurance horses
Source: BMC Vet Res. 2016 Oct 10;12:226. doi: 10.1186/s12917-016-0846-x (PMC5057441; doi:10.1186/s12917-016-0846-x)
Supplement: Additional file 3: — Results of the multivariate regression analyses, showing the influence of external factors considered as independent variables on left atrial (LA) and great vessel echocardiographic dimensions. (DOCX 29 kb) [file 12917_2016_846_MOESM3_ESM.docx]

Additional File 3: Results of the multivariate regression analyses, showing the influence of external factors considered as independent variables on left atrial (LA) and great vessels echocardiographic dimensions.

| Dependent variable | Independent variable | | Effect on dependent variable | 95% -CI | R^2^ of the model | Type of mathematical model |
| --- | --- | --- | --- | --- | --- | --- |
|  | **2-Dimensional measurements** | | | | | |
| **LAD_max_**  (n= 255) | **BWT** | | **0.31 cm increase with each 100kg** | -0.06, 0.68 | **11.2** | Robust linear model |
|  | WH | | nr |  |  |  |
|  | TC | | nr |  |  |  |
|  | BL | | 0.02cm increase for each cm | 0.004, 0.04 |  |  |
|  | breed | 1 | Ref |  |  |  |
|  |  | 2 | 0.21 cm lower then 1 | -0.47, 0.04 |  |  |
|  |  | 3 | nr |  |  |  |
|  |  | 4 | nr |  |  |  |
|  | sex | f | Ref |  |  |  |
|  |  | g | nr |  |  |  |
|  |  | im | nr |  |  |  |
|  | **age** | **4y** | **Ref** |  |  |  |
|  |  | **5y** | **0.27cm higher than 4** | -0.03, 0.58 |  |  |
|  |  | **6y** | **0.37 cm higher than 4** | 0.09, 0.65 |  |  |
|  | Km-career | | nr |  |  |  |
|  | d-career | | nr |  |  |  |
|  |  | | | | | |
| **LAD_a_**  (n= 255) | BWT | | nr |  | **13.1** | Robust linear model |
|  | WH | | nr |  |  |  |
|  | TC | | 0.01 cm increase with each cm | 0.0002, 0.02 |  |  |
|  | BL | | 0.03 cm increase with each cm | 0.01, 0.05 |  |  |
|  | breed | 1 | Ref |  |  |  |
|  |  | 2 | nr |  |  |  |
|  |  | 3 | nr |  |  |  |
|  |  | 4 | nr |  |  |  |
|  | sex | f | Ref |  |  |  |
|  |  | g | 0.32cm lower than F | -0.68, 0.03 |  |  |
|  |  | im | nr |  |  |  |
|  | **age** | **4y** | **Ref** |  |  |  |
|  |  | **5y** | nr |  |  |  |
|  |  | **6y** | **0.54 cm higher than 4** | 0.24, 0.83 |  |  |
|  | Km-career | | nr |  |  |  |
|  | d-career | | nr |  |  |  |
|  |  | | | | | |
| **LAD_min_**  (n= 255) | BWT | | nr |  | **11.1** | Robust linear model |
|  | WH | | 0.05cm increase with each cm | 0.01, 0.09 |  |  |
|  | TC | | nr |  |  |  |
|  | BL | | 0.02cm increase with each cm | 0.001, 0.04 |  |  |
|  | breed | 1 | Ref |  |  |  |
|  |  | 2 | nr |  |  |  |
|  |  | 3 | nr |  |  |  |
|  |  | 4 | nr |  |  |  |
|  | sex | f | Ref |  |  |  |
|  |  | g | 0.49 cm lower than F | -0.88, -0.10 |  |  |
|  |  | im | nr |  |  |  |
|  | **age** | **4y** | **Ref** |  |  |  |
|  |  | **5y** | nr |  |  |  |
|  |  | **6y** | **0.57 cm higher than 4** | 0.24, 0.90 |  |  |
|  | Km-career | | nr |  |  |  |
|  | d-career | | nr |  |  |  |
|  | **Area based measurements** | | | | | |
| **LAA_max_**  (n= 255) | BWT | | nr |  | **15.9** | Robust linear model |
|  | **WH** | | **0.35 cm^2^ increase with each cm** | 0.004, 0.70 |  |  |
|  | **TC** | | **0.49 cm^2^ increase with each cm** | 0.28, 0.70 |  |  |
|  | BL | | nr |  |  |  |
|  | breed | 1 | Ref |  |  |  |
|  |  | 2 | nr |  |  |  |
|  |  | 3 | nr |  |  |  |
|  |  | 4 | nr |  |  |  |
|  | sex | f | Ref |  |  |  |
|  |  | g | nr |  |  |  |
|  |  | im | nr |  |  |  |
|  | **age** | **4y** | **Ref** |  |  |  |
|  |  | **5y** | nr |  |  |  |
|  |  | **6y** | **2,83 cm^2^ higher than 4** | -0.07, 5.74 |  |  |
|  | Km-career | | nr |  |  |  |
|  | d-career | | nr |  |  |  |
|  |  | | | | | |
| **LAA_a_**  (n= 255) | BWT | | nr |  | **16.3** | Logarithmic model |
|  | **WH** | | **0.4% increase with each cm** | -0.1, 1.0 |  |  |
|  | **TC** | | **0.5% increase with each cm** | 0.2, 0.9 |  |  |
|  | **BL** | | **0.3% increase with each cm** | 0.05, 0.6 |  |  |
|  | **breed** | **1** | **Ref** |  |  |  |
|  |  | **2** | **5% lower than 1** | 1, 9 |  |  |
|  |  | **3** | **6% lower than 1** | -2, 13 |  |  |
|  |  | **4** | **9% lower than 1** | -1, 19 |  |  |
|  | **sex** | **f** | nr |  |  |  |
|  |  | **g** | nr |  |  |  |
|  |  | **im** | **6% lower than F** | 0.4, 11 |  |  |
|  | **age** | **4y** | nr |  |  |  |
|  |  | **5y** | nr |  |  |  |
|  |  | **6y** | **4% higher than 4** | -0.6, 9 |  |  |
|  | Km-career | | nr |  |  |  |
|  | d-career | | nr |  |  |  |
|  |  | | | | | |
| **LAA_min_**  (n= 255) | BWT | | nr |  | **11.7** | Logarithmic model |
|  | **WH** | | **0.8% increase with each cm** | 0.1, 1.5 |  |  |
|  | **TC** | | **0.2% increase with each cm** | 0, 0.4 |  |  |
|  | **BL** | | **0.4% increase with each cm** | 0.06, 0.8 |  |  |
|  | **breed** | **1** | **Ref** |  |  |  |
|  |  | **2** | **4% lower than 1** | -0.8, 9 |  |  |
|  |  | **3** | **9% lower than 1** | -2, 17 |  |  |
|  |  | **4** | **10% lower than 1** | -3, 21 |  |  |
|  | **sex** | **f** | **Ref** |  |  |  |
|  |  | **g** | **7% lower than F** | 0, 13 |  |  |
|  |  | **im** | **5% lower than F** | 0.2, 10 |  |  |
|  | **age** | **4y** | nr |  |  |  |
|  |  | **5y** | nr |  |  |  |
|  |  | **6y** | **6% higher than 4** | -0.4, 12 |  |  |
|  | Km-career | | nr |  |  |  |
|  | d-career | | nr |  |  |  |
|  | **Dimension vessels** | | | | | |
| **AOD**  (n= 245) | **BWT** | | **0.11 cm increase with each 100kg** | -0.10, 0.32 | **14.4** | Robust linear model |
|  | WH | | nr |  |  |  |
|  | TC | | 0.02 cm increase with each cm | 0.014, 0.025 |  |  |
|  | BL | | 0.01 cm increase with each cm | 0.001, 0.022 |  |  |
|  | breed | 1 | Ref |  |  |  |
|  |  | 2 | nr |  |  |  |
|  |  | 3 | nr |  |  |  |
|  |  | 4 | nr |  |  |  |
|  | **sex** | **f** | **Ref** |  |  |  |
|  |  | **g** | **0.16 cm higher than in F** | 0.02, 0.31 |  |  |
|  |  | **im** | **0.13 cm higher than in F** | -0.05, 0.32 |  |  |
|  | **age** | **4y** | nr |  |  |  |
|  |  | **5y** | nr |  |  |  |
|  |  | **6y** | **0.2 cm higher than in 4** | 0.05, 0.36 |  |  |
|  | Km-career | | nr |  |  |  |
|  | d-career | | nr |  |  |  |
|  |  | | | | | |
| **PAD**  (n= 239) | **BWT** | | **0.27 cm increase with each 100kg** | 0.02, 0.52 | **17.2** | Robust linear model |
|  | WH | | nr |  |  |  |
|  | TC | | nr |  |  |  |
|  | BL | | nr |  |  |  |
|  | breed | 1 | Ref |  |  |  |
|  |  | 2 | nr |  |  |  |
|  |  | 3 | nr |  |  |  |
|  |  | 4 | nr |  |  |  |
|  | **sex** | **f** | **Ref** |  |  |  |
|  |  | **g** | **0.34 cm lower than F** | -0.51, 0.04 |  |  |
|  |  | **im** | **0.23 cm lower than F** | -0.55, -0.13 |  |  |
|  | **age** | **4y** | **Ref** |  |  |  |
|  |  | **5y** | **0.33 cm lower than 4** | -0.60, -0.05 |  |  |
|  |  | **6y** | nr |  |  |  |
|  | **Km-career** | | **0.11 cm increase with each 100km** | 0.06, 0.15 |  |  |
|  | **d-career** | | **0.0008 cm decrease with each month** | -0.001, -0.0003 |  |  |

Models with relevant R^2^ above 10% are highlighted in bold. 1, Group Purebred Arabians; 2, Group Part-bred Arabians; 3, Group Anglo-Arabians; 4, Group Others; CI, confidence interval; f, females; g, geldings; im, intact males; n, number of measurements available for the analyses; nr, non-relevant in the model; y, years; see abbreviation list for meaning of abbreviations for LA and great vessels measurements.
