# Supplementary material for: Relationships between body dimensions, body weight, age, gender, breed and echocardiographic dimensions in young endurance horses
Source: BMC Vet Res. 2016 Oct 10;12:226. doi: 10.1186/s12917-016-0846-x (PMC5057441; doi:10.1186/s12917-016-0846-x)
Supplement: Additional file 4: — Results of the multivariate regression analyses, showing the influence of external factors considered as independent variables on left ventricular (LV) echocardiographic dimensions. (DOCX 37 kb) [file 12917_2016_846_MOESM4_ESM.docx]

Additional File 4: Results of the multivariate regression analyses, showing the influence of external factors considered as independent variables on left ventricle (LV) echocardiographic dimensions.

| Dependent variable | Independent variable | | Effect on dependent variable | 95% -CI | R^2^ of the model | Type of mathematical model |
| --- | --- | --- | --- | --- | --- | --- |
|  | **2-Dimensional measurements** | | | | | |
| **IVS_d_**  (n= 266) | BWT | | 12% increase with each 100kg | 6, 19 | 5.9 | Logarithmic model |
|  | WH | | nr |  |  |  |
|  | TC | | 0.1% decrease with each cm | 0, 0.2 |  |  |
|  | BL | | 0.3% decrease with each cm | 0, 0.6 |  |  |
|  | breed | 1 | Ref |  |  |  |
|  |  | 2 | 3% lower than 1 | -0.8, 7 |  |  |
|  |  | 3 | 4% higher than 1 | -3, 11 |  |  |
|  |  | 4 | 6% higher than 1 | -3, 16 |  |  |
|  | sex | f | Ref |  |  |  |
|  |  | g | nr |  |  |  |
|  |  | im | nr |  |  |  |
|  | age | 4y | Ref |  |  |  |
|  |  | 5y | nr |  |  |  |
|  |  | 6y | nr |  |  |  |
|  | Km-career | | nr |  |  |  |
|  | d-career | | 0.1% increase with each months |  |  |  |
|  |  | | | | | |
| **LVID_d_**  (n= 261) | BWT | | increase until a weight of 490kg  decrease from a weight of 490kg | na | 7.2 | Robust logarithmic model |
|  | WH | | nr |  |  |  |
|  | TC | | nr |  |  |  |
|  | BL | | nr |  |  |  |
|  | breed | 1 | Ref |  |  |  |
|  |  | 2 | 3% lower than 1 | 0.3, 5 |  |  |
|  |  | 3 | 4% higher than 1 | -0.5, 8 |  |  |
|  |  | 4 | 5% lower than 1 | 0.3, 10 |  |  |
|  | sex | f | Ref |  |  |  |
|  |  | g | nr |  |  |  |
|  |  | im | 1% higher than F | -1, 3 |  |  |
|  | age | 4y | Ref |  |  |  |
|  |  | 5y | nr |  |  |  |
|  |  | 6y | nr |  |  |  |
|  | Km-career | | nr |  |  |  |
|  | d-career | | nr |  |  |  |
|  |  | | | | | |
| **LVFW_d_**  (n= 261) | BWT | | decrease until a weight of 402kg  increase from a weight of 402kg | na | 7.1 | Logarithmic model |
|  | WH | | nr |  |  |  |
|  | TC | | nr |  |  |  |
|  | BL | | 0,4% increase with each cm | 0, 0.9 |  |  |
|  | breed | 1 | Ref |  |  |  |
|  |  | 2 | nr |  |  |  |
|  |  | 3 | nr |  |  |  |
|  |  | 4 | nr |  |  |  |
|  | sex | f | Ref |  |  |  |
|  |  | g | nr |  |  |  |
|  |  | im | nr |  |  |  |
|  | age | 4y | Ref |  |  |  |
|  |  | 5y | nr |  |  |  |
|  |  | 6y | nr |  |  |  |
|  | Km-career | | 2% increase with each 100km | 0.8, 3 |  |  |
|  | d-career | | nr |  |  |  |
|  |  | | | | | |
| **MWT**  (n= 257) | BWT | | decrease until a weight of 368kg  increase from a weight of 368kg | na | 9 | Logartimic model |
|  | WH | | nr |  |  |  |
|  | TC | | nr |  |  |  |
|  | BL | | nr |  |  |  |
|  | breed | 1 | Ref |  |  |  |
|  |  | 2 | nr |  |  |  |
|  |  | 3 | nr |  |  |  |
|  |  | 4 | 8% higher than 1 | -0.4, 18 |  |  |
|  | sex | f | Ref |  |  |  |
|  |  | g | nr |  |  |  |
|  |  | im | nr |  |  |  |
|  | age | 4y | Ref |  |  |  |
|  |  | 5y | nr |  |  |  |
|  |  | 6y | nr |  |  |  |
|  | Km-career | | 1% increase with each 100km | 0.5, 2 |  |  |
|  | d-career | | nr |  |  |  |
|  |  | | | | | |
| **RWT**  (n= 257) | BWT | | decrease until a weight of 415kg  increase from a weight of 415kg | na | 4 | Logarithmic model |
|  | WH | | nr |  |  |  |
|  | TC | | nr |  |  |  |
|  | BL | | nr |  |  |  |
|  | breed | 1 | Ref |  |  |  |
|  |  | 2 | nr |  |  |  |
|  |  | 3 | nr |  |  |  |
|  |  | 4 | 15% higher to 1 | 2, 29 |  |  |
|  | sex | f | Ref |  |  |  |
|  |  | g | nr |  |  |  |
|  |  | im | nr |  |  |  |
|  | age | 4y | Ref |  |  |  |
|  |  | 5y | nr |  |  |  |
|  |  | 6y | nr |  |  |  |
|  | Km-career | | 1% increase witch each 100km | 0.3, 2 |  |  |
|  | d-career | | nr |  |  |  |
|  |  | | | | | |
| **LV_mass_**  (n= 260) | **BWT** | | **19% increase with each 100kg** | 12, 26 | **21.4** | Logarithmic model |
|  | WH | | nr |  |  |  |
|  | TC | | nr |  |  |  |
|  | BL | | nr |  |  |  |
|  | **breed** | 1 | Ref |  |  |  |
|  |  | **2** | **5% lower than 1** | 1, 10 |  |  |
|  |  | **3** | **7% higher than 1** | -1, 15 |  |  |
|  |  | 4 | nr |  |  |  |
|  | sex | f | Ref |  |  |  |
|  |  | g | nr |  |  |  |
|  |  | im | nr |  |  |  |
|  | age | 4y | Ref |  |  |  |
|  |  | 5y | nr |  |  |  |
|  |  | 6y | nr |  |  |  |
|  | **Km-career** | | **1% increase with each 100km** | 0.7, 2 |  |  |
|  | d-career | | nr |  |  |  |
|  | **Area based measurements** | | | | | |
| **LVIL_d_**  (n= 317) | BWT | | 0.63 cm increase with each 100kg | 0.04, 1.21 | 6 | Robust linear model |
|  | WH | | 0.07 cm increase with each cm | 0.02, 0.12 |  |  |
|  | TC | | nr |  |  |  |
|  | BL | | 0.03 cm decrease with each cm | -0.05, -0.01 |  |  |
|  | breed | 1 | Ref |  |  |  |
|  |  | 2 | nr |  |  |  |
|  |  | 3 | nr |  |  |  |
|  |  | 4 | nr |  |  |  |
|  | sex | f | Ref |  |  |  |
|  |  | g | nr |  |  |  |
|  |  | im | nr |  |  |  |
|  | age | 4y | Ref |  |  |  |
|  |  | 5y | 0.21cm higher than 4 | -0.21, 0.63 |  |  |
|  |  | 6y | 0.45 cm higher than 4 | -0.04, 0.95 |  |  |
|  | Km-career | | 0.06 cm decrease with each 100km | -0.14, 0.02 |  |  |
|  | d-career | | nr |  |  |  |
|  |  | | | | | |
| **LVIA_d_**  (n= 317) | **BWT** | | **0.17 cm^2^ increase with each 100kg** | 0.10, 0.24 | **15.4** | Robust linear model |
|  | **WH** | | **0.91 cm^2^ increase with each cm** | 0.30, 1.52 |  |  |
|  | TC | | nr |  |  |  |
|  | BL | | 0.45 cm^2^ decrease with each cm | -0.77, -0.12 |  |  |
|  | breed | 1 | Ref |  |  |  |
|  |  | 2 | nr |  |  |  |
|  |  | 3 | nr |  |  |  |
|  |  | 4 | nr |  |  |  |
|  | sex | f | Ref |  |  |  |
|  |  | g | 3.81 cm^2^ lower than F | -9.20, 1.58 |  |  |
|  |  | im | 5,38 cm^2^ lower than F | -9.84, -0.92 |  |  |
|  | age | 4y | Ref |  |  |  |
|  |  | 5y | nr |  |  |  |
|  |  | 6y | nr |  |  |  |
|  | **Km-career** | | **0.75 cm^2^ increase with each 100km** |  |  |  |
|  | d-career | | nr | -0.01, 1.57 |  |  |
|  |  | | | | | |
| **LVIV_d_**  (n= 312) | **BWT** | | **199 ml increase with each 100kg** | 121.4, 277.9 | **17.3** | Robust linear model |
|  | **WH** | | **8.49 ml increase with each cm** | 1.82, 15.15 |  |  |
|  | TC | | nr |  |  |  |
|  | BL | | 4.24 ml decrease with each cm | -7.82, -0.66 |  |  |
|  | breed | 1 | Ref |  |  |  |
|  |  | 2 | nr |  |  |  |
|  |  | 3 | nr |  |  |  |
|  |  | 4 | nr |  |  |  |
|  | sex | f | Ref |  |  |  |
|  |  | g | nr |  |  |  |
|  |  | im | nr |  |  |  |
|  | age | 4y | Ref |  |  |  |
|  |  | 5y | nr |  |  |  |
|  |  | 6y | nr |  |  |  |
|  | **Km-career** | | **12 ml increase with each 100km** | 0.03, 0.20 |  |  |
|  | d-career | | nr |  |  |  |
|  |  | | | | | |
| **SV**  (n= 317) | **BWT** | | **21% increase with each 100kg** | 13, 30 | **14.2** | Logarithmic model |
|  | **WH** | | **0.6% increase with each cm** | 0, 1.3 |  |  |
|  | TC | | nr |  |  |  |
|  | BL | | 0.4% decrease with each cm | 0.1, 0.7 |  |  |
|  | breed | 1 | Ref |  |  |  |
|  |  | 2 | nr |  |  |  |
|  |  | 3 | nr |  |  |  |
|  |  | 4 | nr |  |  |  |
|  | sex | f | Ref |  |  |  |
|  |  | g | nr |  |  |  |
|  |  | im | nr |  |  |  |
|  | age | 4y | Ref |  |  |  |
|  |  | 5y | nr |  |  |  |
|  |  | 6y | nr |  |  |  |
|  | Km-career | | nr |  |  |  |
|  | d-career | | nr |  |  |  |
|  |  | | | | | |
| **CO**  (n= 317) | BWT | | 17 % increase with each 100kg | 5, 30 | 5.9 | Logarithmic model |
|  | WH | | 0.9% increase with each cm | 0, 1.9 |  |  |
|  | TC | | nr |  |  |  |
|  | BL | | 0.5% decrease with each cm | 0, 1 |  |  |
|  | breed | 1 | Ref |  |  |  |
|  |  | 2 | - nr |  |  |  |
|  |  | 3 | nr |  |  |  |
|  |  | 4 | nr |  |  |  |
|  | sex | f | Ref |  |  |  |
|  |  | g | nr |  |  |  |
|  |  | im | nr |  |  |  |
|  | age | 4y | Ref |  |  |  |
|  |  | 5y | nr |  |  |  |
|  |  | 6y | nr |  |  |  |
|  | Km-career | | nr |  |  |  |
|  | d-career | | nr |  |  |  |

Models with relevant R^2^ above 10% are highlighted in bold. 1, Group Purebred Arabians; 2, Group Part-bred Arabians; 3, Group Anglo-Arabians; 4, Group Others; CI, confidence interval; f, females; g, geldings; im, intact males; n, number of measurements available for the analysis; na, not applicable; nr, non-relevant in the model; y, years; see abbreviation list for meaning of abbreviations for LV measurements.

Models with additionally introduced square terms for the body weight (dependent variable: LVID_d_, LVFW_d_, MWT, RWT): no regression coefficients and confidence intervals were indicated since for quadratic functions, slopes and thus regressions coefficients differ with different values of the considered predictors.
