# Supplementary material for: Relationships between body dimensions, body weight, age, gender, breed and echocardiographic dimensions in young endurance horses
Source: BMC Vet Res. 2016 Oct 10;12:226. doi: 10.1186/s12917-016-0846-x (PMC5057441; doi:10.1186/s12917-016-0846-x)
Supplement: Additional file 5: — Results of the ANOVAs and Student’s t-tests reporting p-values for comparisons of morphometric measurements between age groups, genders and breeds groups. (DOCX 17 kb) [file 12917_2016_846_MOESM5_ESM.docx]

Additional File 5: Results of the ANOVAs and Student’s t-tests reporting p-values for comparisons of morphometric measurements between age groups, genders and breed groups.

|  | BWT | BSA | WH | TC | BL | d-career | Km-career |
| --- | --- | --- | --- | --- | --- | --- | --- |
| Comparison |  | | | | | | |
| *-overall ages* | **0.004** | **0.003** | 0.161 | 0.177 | 0.164 | **< 0.0001** | **< 0.0001** |
| *-Group 4y vs.*  *Group 5y* | **0.004** | **0.003** | 0.491 | 0.364 | 0.619 | **< 0.0001** | **0.003** |
| *-Group 5y vs.*  *Group 6y* | **0.030** | **0.030** | 0.159 | 0.194 | 0.185 | **< 0.0001** | **< 0.001** |
| *-Group 4y vs.*  *Group 6y* | 0.299 | 0.288 | 0.491 | 0.649 | 0.362 | **< 0.0001** | **< 0.0001** |
|  |  |  |  |  |  |  |  |
| *-overall genders* | 0.115 | 0.128 | 0.051 | **0.007** | 0.081 | 0.233 | 0.442 |
| *-Females vs.*  *Intact males* | 0.467 | 0.477 | 0.726 | **0.007** | 0.291 | 0.291 | 0.551 |
| *-Females vs. Geldings* | 0.224 | 0.244 | 0.086 | 0.179 | 0.291 | 0.982 | 0.979 |
| *-Geldings vs.*  *Intact males* | 0.152 | 0.170 | 0.086 | 0.179 | 0.082 | 0.291 | 0.551 |
| *-Females vs.*  *all males* | 0.437 | 0.453 | 0.161 | **0.007** | 0.635 | 0.439 | 0.588 |
|  |  |  |  |  |  |  |  |
| *-overall breeds* | **< 0.0001** | **< 0.0001** | **0.001** | **< 0.0001** | **0.002** | 0.502 | 0.359 |
| *-Purebred Arabians vs. Part-bred Arabians* | **0.0002** | **0.0002** | **0.021** | **< 0.0001** | **0.021** | 0.937 | 0.863 |
| *- Purebred Arabians vs. Anglo-Arabians* | **< 0.0001** | **< 0.0001** | **0.020** | **0.006** | 0.102 | 0.937 | 0.801 |
| *- Purebred Arabians vs. Others* | **0.005** | **0.006** | 0.107 | **0.031** | 0.111 | 0.621 | 0.512 |
| *-Part-bred Arabians vs. Anglo-Arabians* | 0.077 | 0.077 | 0.513 | 0.906 | 0.797 | 0.937 | 0.863 |
| *-Par-bred Arabians vs. Others* | 0.307 | 0.318 | 0.631 | 0.906 | 0.797 | 0.643 | 0.621 |
| *-Anglo-Arabians vs. Others* | 0.773 | 0.759 | 0.887 | 0.971 | 0.797 | 0.621 | 0.863 |

Significant p-values are highlighted in bold. See abbreviation list for meaning of abbreviations for morphometric measurements.
