# Supplementary material for: Relationships between body dimensions, body weight, age, gender, breed and echocardiographic dimensions in young endurance horses
Source: BMC Vet Res. 2016 Oct 10;12:226. doi: 10.1186/s12917-016-0846-x (PMC5057441; doi:10.1186/s12917-016-0846-x)
Supplement: Additional file 6: — Results of the ANOVAs and Student’s t-tests reporting p-values for comparisons of left atrial (LA) and great vessels dimensions between age groups, genders and breeds groups. (DOCX 18 kb) [file 12917_2016_846_MOESM6_ESM.docx]

Additional File 6: Results of the ANOVAs and Student’s t-tests reporting p-values for comparisons of left atrial (LA) and great vessels dimensions between age groups, genders and breed groups.

|  | LAD_max_ | LAD_a_ | LAD_min_ | LAA_max_ | LAA_a_ | LAA_min_ | Active  LA FAC | Passive LA FAC | active:total LA AC | AOD | PA | AO/PAD |
| --- | --- | --- | --- | --- | --- | --- | --- | --- | --- | --- | --- | --- |
| Comparison |  | | | | | | | | | | | |
| *-Overall ages* | **0.002** | **0.002** | **0.005** | 0.064 | 0.050 | 0.170 | 0.582 | 0.366 | 0.549 | **0.023** | **0.038** | **0.001** |
| *-Group 4y vs.*  *Group 5y* | **0.008** | 0.143 | 0.190 | 0.666 | 0.700 | 0.574 | 0.717 | 0.504 | 0.809 | 0.497 | **0.032** | **0.010** |
| *-Group 5y vs.*  *Group 6y* | 0.953 | 0.143 | 0.190 | 0.089 | 0.057 | 0.574 | 0.717 | 0.818 | 0.621 | 0.131 | 0.281 | 0.716 |
| *-Group 4y vs.*  *Group 6y* | **0.005** | **0.002** | **0.004** | 0.151 | 0.118 | 0.168 | 0.978 | 0.440 | 0.809 | **0.026** | 0.281 | **0.002** |
|  |  | | | | | | | | | | | |
| *-Overall genders* | 0.083 | **0.017** | **0.021** | 0.219 | **0.008** | **0.013** | 0.067 | **0.027** | 0.171 | 0.161 | **0.007** | **0.001** |
| *-Females vs.*  *Intact males* | 0.202 | **0.021** | **0.021** | 0.244 | **0.006** | **0.037** | 0.879 | **0.022** | 0.391 | 0.671 | 0.154 | 0.214 |
| *-Females vs. Geldings* | 0.360 | 0.726 | 0.784 | 0.587 | 0.166 | **0.039** | 0.075 | 0.344 | 0.391 | 0.164 | **0.007** | **0.001** |
| *-Geldings vs.*  *Intact males* | 0.076 | **0.021** | **0.036** | 0.587 | 0.113 | 0.546 | 0.221 | 0.126 | 0.193 | 0.487 | 0.509 | 0.174 |
| *-Females vs.*  *All males* | 0.933 | 0.348 | 0.144 | 0.139 | **0.014** | **0.037** | 0.087 | 0.051 | 0.639 | 0.112 | **0.002** | **0.001** |
|  |  | | | | | | | | | | | |
| *-Overall breeds* | 0.238 | 0.223 | 0.632 | 0.379 | 0.291 | 0.230 | 0.335 | 0.285 | 0.831 | 0.695 | 0.613 | 0.639 |
| *- Purebred Arabians vs. Part-bred Arabians* | 0.598 | 0.726 | 0.895 | 0.932 | 0.915 | 0.653 | 0.623 | 0.704 | 0.979 | 0.948 | 0.895 | 0.920 |
| *- Purebred Arabians vs. Anglo-Arabians* | 0.598 | 0.345 | 0.895 | 0.702 | 0.915 | 0.904 | 0.799 | 0.785 | 0.967 | 0.902 | 0.904 | 0.908 |
| *- Purebred Arabians vs. Others* | 0.671 | 0.620 | 0.895 | 0.702 | 0.337 | 0.371 | 0.899 | 0.547 | 0.985 | 0.948 | 0.917 | 0.920 |
| *- Part-bred Arabians vs. Anglo-Arabians* | 0.365 | 0.341 | 0.895 | 0.702 | 0.915 | 0.904 | 0.968 | 0.803 | 0.982 | 0.948 | 0.809 | 0.821 |
| *- Par-bred Arabians vs. Others* | 0.786 | 0.620 | 0.895 | 0.702 | 0.408 | 0.663 | 0.968 | 0.785 | 0.985 | 0.948 | 0.914 | 0.920 |
| *- Anglo-Arabians vs. Others* | 0.520 | 0.258 | 0.727 | 0.400 | 0.408 | 0.653 | 0.968 | 0.803 | 0.985 | 0.948 | 0.916 | 0.920 |

Significant p-values are highlighted in bold. See abbreviation list for meaning of abbreviations for LA and great vessels measurements.
