# Supplementary material for: Relationships between body dimensions, body weight, age, gender, breed and echocardiographic dimensions in young endurance horses
Source: BMC Vet Res. 2016 Oct 10;12:226. doi: 10.1186/s12917-016-0846-x (PMC5057441; doi:10.1186/s12917-016-0846-x)
Supplement: Additional file 7: — Results of the ANOVAs and Student’s t-tests reporting p-values for comparisons of left ventricular (LV) echocardiographic dimensions between age groups, genders and breeds groups. (DOCX 21 kb) [file 12917_2016_846_MOESM7_ESM.docx]

Additional File 7: Results of the ANOVAs and Student’s t-tests reporting p-values for comparisons of left ventricular (LV) echocardiographic dimensions between age groups, genders and breed groups.

|  | LVID_d_ | LVID_s_ | IVS_d_ | IVS_s_ | LVFW_d_ | LVFW_s_ | LVIL_d_ | LVIL_s_ | LVIA_d_ | LVIA_s_ | LVIV_d_ | LVIV_s_ | LV FS | LV FAC | MWR | RWT | LV_mass_ | SV | CO |
| --- | --- | --- | --- | --- | --- | --- | --- | --- | --- | --- | --- | --- | --- | --- | --- | --- | --- | --- | --- |
| Comparison |  | | | | | | | | | | | | | | | | | | |
| *-overall ages* | **0.045** | **0.016** | 0.183 | 0.468 | 0.268 | 0.218 | 0.070 | **0.010** | **0.003** | **0.002** | **0.003** | **0.004** | 0.149 | 0.195 | 0.112 | 0.334 | **0.002** | 0.070 | 0.60 |
| *-Group 4y vs.*  *Group 5y* | **0.038** | **0,018** | 0.283 | 0.740 | 0.986 | 0.745 | 0.073 | 0.097 | **0.033** | **0.011** | 0.064 | **0.027** | 0.225 | 0.287 | 0.419 | 0.678 | **0.006** | 0.363 | 0.725 |
| *-Group 5y vs.*  *Group 6y* | 0.295 | 0.561 | 0.758 | 0.740 | 0.405 | 0.269 | 0.868 | 0.317 | 0.344 | 0.670 | 0.234 | 0.464 | 0.904 | 0.921 | 0.347 | 0.383 | 0.803 | 0.363 | 0.947 |
| *-Group 4y vs.*  *Group 6y* | 0.295 | 0.060 | 0.226 | 0.524 | 0.405 | 0.351 | 0.073 | **0.008** | **0.002** | **0.003** | **0.002** | **0.004** | 0.225 | 0.287 | 0.112 | 0.532 | **0.004** | 0.062 | 0.735 |
|  |  | | | | | | | | | | | | | | | | | | |
| *-overall genders* | 0.159 | 0.899 | 0.173 | 0.273 | 0.915 | 0.565 | 0.378 | 0.381 | 0.574 | 0.827 | 0.762 | 0.649 | 0.467 | 0.957 | 0.577 | 0.384 | 0.247 | 0.924 | 0.232 |
| *-Females vs.*  *Intact males* | 0.809 | 0.968 | 0.191 | 0.355 | 0.967 | 0.750 | 0.445 | 0.922 | 0.709 | 0.926 | 0.871 | 0.727 | 0.975 | 0.993 | 0.682 | 0.620 | 0.464 | 0.979 | 0.481 |
| *-Females vs. Geldings* | 0.175 | 0.968 | 0.779 | 0.889 | 0.967 | 0.750 | 0.661 | 0.456 | 0.709 | 0.926 | 0.871 | 0.789 | 0.573 | 0.993 | 0.851 | 0.620 | 0.309 | 0.979 | 0.406 |
| *-Geldings vs.*  *Intact males* | 0.365 | 0.996 | 0.243 | 0.355 | 0.967 | 0.651 | 0.661 | 0.556 | 0.749 | 0.916 | 0.871 | 0.749 | 0.593 | 0.993 | 0.682 | 0.439 | 0.831 | 0.999 | 0.281 |
| *-Females vs.*  *All males* | 0.151 | 0.644 | 0.271 | 0.543 | 0.686 | 0.826 | 0.215 | 0.318 | 0.315 | 0.554 | 0.487 | 0.518 | 0.406 | 0.771 | 0.756 | 0.784 | 0.097 | 0.691 | 0.605 |
|  |  | | | | | | | | | | | | | | | | | | |
| *-overall breeds* | **0.010** | 0.175 | **0.015** | 0.328 | 0.200 | 0.486 | 0.443 | 0.516 | 0.126 | 0.803 | 0.072 | 0.922 | 0.859 | 0.194 | 0.096 | 0.056 | **0.007** | **0.018** | 0.343 |
| *- Purebred Arabians vs. Part-bred Arabians* | 0.249 | 0.563 | 0.206 | 0.949 | 0.532 | 0.822 | 0.879 | 0.758 | 0.800 | 0.966 | 0.715 | 0.999 | 0.961 | 0.716 | 0.758 | 0.851 | 0.823 | 0.689 | 0.781 |
| *- Purebred Arabians vs. Anglo-Arabians* | 0.152 | 0.569 | 0.206 | 0.409 | 0.788 | 0.876 | 0.622 | 0.910 | 0.174 | 0.973 | 0.085 | 0.999 | 0.998 | 0.243 | 0.422 | 0.921 | **0.008** | **0.015** | 0.484 |
| *- Purebred Arabians vs. Others* | 0.108 | 0.569 | 0.518 | 0.968 | 0.376 | 0.822 | 0.879 | 0.910 | 0.731 | 0.960 | 0.715 | 0.983 | 0.998 | 0.929 | 0.179 | **0.037** | 0.823 | 0.689 | 0.928 |
| *- Part-bred Arabians vs. Anglo-Arabians* | 0.052 | 0.355 | **0.031** | 0.343 | 0.844 | 0.822 | 0.583 | 0.996 | 0.209 | 0.973 | 0.194 | 0.999 | 0.998 | 0.715 | 0.527 | 0.967 | **0.004** | 0.087 | 0.781 |
| *-Part-bred Arabians vs. Others* | 0.249 | 0.609 | 0.206 | 0.996 | 0.758 | 0.876 | 0.879 | 0.996 | 0.731 | 0.972 | 0.676 | 0.983 | 0.988 | 0.929 | 0.273 | 0.099 | 0.823 | 0.689 | 0.822 |
| *-Anglo-Arabians vs. Others* | **0.014** | 0.363 | 0.687 | 0.572 | 0.758 | 0.822 | 0.622 | 0.996 | 0.174 | 0.972 | 0.139 | 0.983 | 0.998 | 0.715 | 0.758 | 0.196 | 0.204 | 0.087 | 0.781 |

Significant p-values are highlighted in bold. See abbreviation list for meaning of abbreviations for LV measurements.
